# A novel CTLA-4 blocking strategy based on nanobody enhances the activity of dendritic cell vaccine-stimulated antitumor cytotoxic T lymphocytes

**DOI:** 10.1038/s41419-023-05914-w

**Published:** 2023-07-07

**Authors:** Wenli Yang, Yanyang Pang, Xi Wang, Zhiheng Lai, Yanda Lu, Shaojiang Zheng, Wu Wang

**Affiliations:** 1 Public Research Center of Hainan Medical University, Hainan Medical University, Haikou, 570100 China; 2 Tumor Institute, The First Affiliated Hospital of Hainan Medical University, Haikou, 570102 China; 3 Key Laboratory of Emergency and Trauma of Ministry of Education, Key Laboratory of Tropical Cardiovascular Diseases Research of Hainan Province, Hainan Women and Children’s Medical Center, Hainan Medical University, Haikou, 571199 China; 4 Department of Anatomy, Zunyi Medical University, Zunyi, 563006 China; 5 School of Traditional Chinese Medicine, Hainan Medical University, Haikou, 570100 China; 6 Guangxi Key Laboratory of Nanobody Research, Guangxi Medical University, Nanning, 530021 China; 7 Department of Anesthesiology, Haikou Third People’s Hospital, Haikou, 570100 China; 8 Department of Anorectal, Hainan Province Hospital of Traditional Chinese Medicine, Haikou, 570100 China

**Keywords:** Tumour immunology, Preclinical research

## Abstract

Despite the great success of CTLA-4 blocking in cancer treatment, the use of anti-CTLA-4 monoclonal antibodies still faces many limitations. Now, immune checkpoint blocking coupled with adoptive cell therapy is gaining much attention. In this paper, we reported a strategy on the basis of anti-CTLA-4 nanobody (Nb)-modified liposomes to improve these obstacles. An Nb36/liposome complex was constructed and utilized as a blocker of the CTLA-4/B7 signal pathway in a combination with dendritic cell (DC)/tumor fusion vaccine to enhance the CD8^+^ T cell cytokine secretion, activation, proliferation, as well as specific cytotoxicity. Moreover, the CD8^+^ T cells induced by LPS-Nb36 and DC/tumor fusion vaccine led to higher CD8^+^ T cell effector function in vivo, which significantly retarded tumor growth and lengthened survival of tumor-bearing mice (HepG2, A549, and MGC-803). Our data demonstrate that the anti-CTLA-4 Nb-modified liposomes in connection with DC/tumor fusion vaccines enhance the CD8^+^ T cell antitumor activity in vitro and in vivo, and is expected to be an alternative therapy for patients with malignancies that have T cell dysfunction or have poor treatment against anti-CTLA-4 mAb.

## Introduction

Adoptive immunotherapy achieves beneficial effects through modulating the regression of tumor cells, which depends on the activation, targeting, as well as persistence of antitumor cytotoxic T cells, whose reaction is restricted by the tumor cells’ properties to evolve multiple strategies to escape immune eradication [1]. T cells are pivotal mediators of the immunoreaction and exert functions in nearly all disease defense processes. In fact, the T-cell immune response to carcinoma can be enhanced by various immunostimulatory methods. Multiple trials indicated that infused autologous tumor-specific cytotoxic T lymphocytes (CTLs) are capable of specifically identifying and dispeling tumor cells. The most popular one is the anticancer vaccines’ development. In this vaccine, tumor antigens are injected into noduliferous animals, resulting in the generation of tumor-specific cytotoxic T cells capable of detecting and killing tumor cells at local and diffuse sites [2, 3]. Dendritic cells (DCs) are forceful antigen-presenting cells (APCs) that act in the stimulation of antitumor immunity and have attracted a great deal of attention [4]. A variety of regimens have been created to transmit tumor-associated antigens (TAAs) to DCs as cancer vaccines, among which DCs infused with whole tumor cells for the generation of DC-tumor fusion cells (DC-tumor fusion vaccine) is an alternative regimen for the therapy of cancer patients [5, 6].

DCs are chemically or physically infused with tumor cells to generate a heterokaryon, which connects DC-derived costimulatory molecules, a large number of tumor-derived antigens, and efficient antigen-processing and presentation machinery [7–9]. Therefore, the DC-tumor fusion vaccine combines the necessary components for the development and presentation of tumor antigens to the host immune cells and the induction of a potent antitumor response. Fusion cells can present various tumor antigens, thereby enhancing the frequency of responding T cells and improving antitumor immunity to the maximum extent, and are therefore considered antitumor immunotherapy with high potential [10]. In our previous investigation, we developed an efficient approach to produce DC/tumor-associated fibroblasts-fusion cells, and tumor-specific CD8^+^ T cells were successfully induced and substantial antitumor responses were achieved in preclinical in vitro and in vivo through this approach [11]. Nevertheless, it was observed in solid tumor therapy that immunosuppression of the tumor microenvironment led to a limitation of effector T cell cytotoxicity and proliferation in vivo [12]. The complicated tumor microenvironment is advantageous to tumor growth and results in the depletion of infused effector T cells, and is thus considered the chief mechanism restricting the curative effect of DC-tumor fusion vaccine therapy, which ultimately limits its clinical application [13].

Numerous regulatory mechanisms exist between immune and tumor cells during tumor occurrence and development. Some of these mechanisms activate the immune system to fight the tumor, while the other has a negative impact on the function of immune cells, thereby boosting tumor cell growth and metastasis, with immune checkpoints (CTLA-4/B7, PD-1/PD-L1, TIM-3, etc.) playing a pivotal role [14]. Among them, cytotoxic T-lymphocyte-associated antigen-4 (CTLA-4) and CTLA-4 ligand (B7-1 and B7-2, also known as CD80 and CD86), acting as a vital immune checkpoint, have attracted extensive attention and shown an obvious clinical activity [15, 16]. CTLA-4 is a protein receptor that is majorly expressed on the activated and regulatory T cells’ surface. When the specific binding of CTLA-4 to CD80 or CD86 is enhanced, it competes with the CD28 for binding to other significant costimulatory factors and inhibits their formation of costimulatory signals, thereby restraining T cell proliferation, activation, together with cell cycle [17]. This results in reduced generation of cytokines (e.g. IL-2, IL-4, and IFN-γ) and reduced levels of IL-2 receptors, ultimately blazing a way in tumors to evade immune surveillance [18, 19]. As the mechanism of this protein is gradually revealed, many studies have disclosed its powerful suppressive role in regulating T cell activity, and a variety of CTLA-4 monoclonal antibodies, such as ipilimumab and tremelimumab, have been recommended by the US FDA for clinical trials and are moving towards clinical application in some cancers, suggesting that a major moment in cancer immunotherapy has emerged [20, 21]. Studies have exhibited promising outcomes concerning the DC-tumor fusion vaccine therapy of diverse cancer types through the blockade of CTLA-4 [22], which signifies that targeting immune inhibitory molecules is able to impede T cell-mediated antitumor immunity. Despite this success, CTLA-4 mAb still faces numerous challenges, including: (1) The excessive molecular weight (160–170 kD) limits the penetration of mAb into tumor microenvironment and the crossing of the blood-brain barrier. Moreover, this makes it difficult to connect to the vector or probe molecules, thus limiting its further development. (2) The mAb non-specifically binds to normal tissues and heterogeneous tumor antigens. (3) The mAb has high production cost due to the complex production process [23]. As a result, developing new antibodies with high efficiency and low cost is necessary to achieve better antitumor treatment outcomes.

Nanobody (Nb) is an antigen-binding fragment of the smallest molecular weight from camelid animals [24]. These animals naturally have an exceptional single-chain antibody (in short of the light chain and CH1), and Nb is acquired after cloning its variable areas that maintain intact antigen-binding capacity, molecular structure, specificity, as well as function. Compared with mAb, Nbs has stronger antigen recognition and binding ability and can bind some less accessible epitopes [25]. These inherent advantages give Nbs great potential value in the diagnosis and therapy of malignant tumors and begin to become a research hotspot in this field. In our previous study, an Nb targeting CTLA-4 (Nb36) was screened by using phage display technology. These anti-CTLA-4 Nb have been demonstrated to efficiently bind CTLA-4 epitopes on activated T cells in vitro [26, 27]. Therefore, we hypothesized that the anti-CTLA-4 Nb could act as a highly potent CTLA-4 signal blocker and achieve similar effects as the anti-CTLA-4 mAb. Although most studies have reported that Nbs have significantly shorter half-life in vivo than monoclonal antibodies [28], more importantly, Nbs have many inherent advantages that have given them great potential for development.

**Fig. 1:**
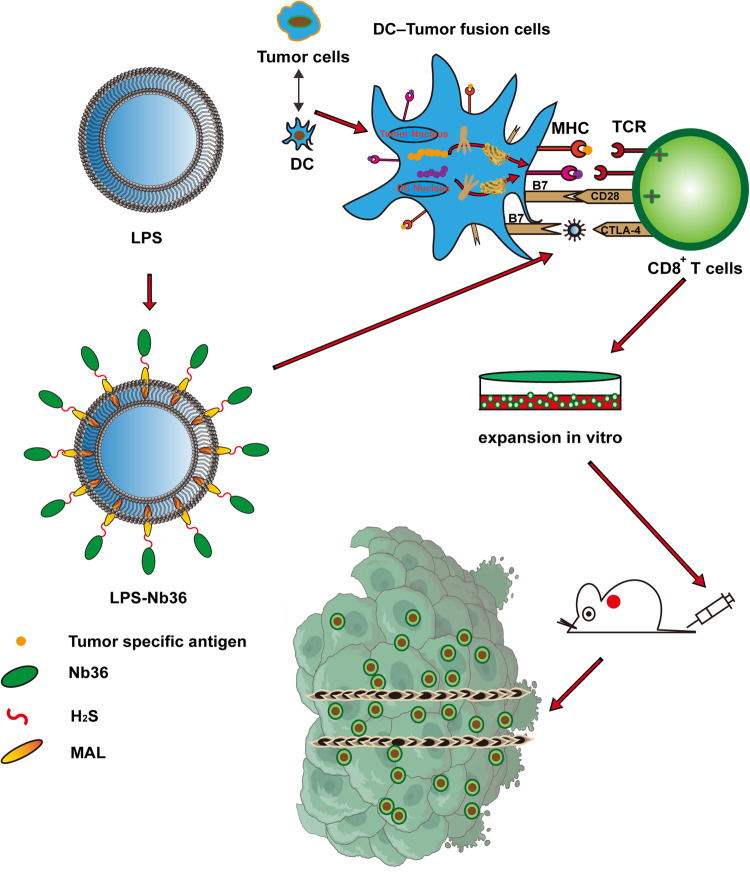
Therapy strategy with DC/tumor fusion+LPS-Nb36 treatment. DC-tumor fusion vaccine were first generated. Then, the Nb36/liposome complex eliminated immunosuppression via blocking CTLA-4-mediated negative co-stimulation in the CD8^+^ T cells stimulated by DC/tumor fusion vaccine.

In this study, we constructed an Nb36/liposome complex (LPS-Nb36) with enhanced antigen-binding ability because of its high concentration of anti-CTLA-4 Nb on the liposomes (Fig. 1). Liposome is a spherical microencapsulated carrier composed of cholesterol and phospholipid. In addition to drug encapsulation, the liposome can also be used as a vector to modify receptors, antibodies, and other targeted substances, thus forming enrichment on their surface [29]. Herein, this work initially discusses the generation of a promising liposome modified with an anti-CTLA-4 Nb as a novel blocker of CTLA-4 molecules. We explored the antitumor function and mechanism of tumor-specific CD8^+^ T lymphocytes induced by DC-tumor fusion vaccine upon stimulation with LPS-Nb36. We aimed to suppress the immunosuppressive signaling from the checkpoint CTLA-4/B7 axis using LPS-Nb36 and induce more efficient tumor-specific CTLs using the comprehensive DC-tumor fusion vaccine technology, thereby achieving superior antitumor functions of adoptive immunotherapy.

## Materials and methods

### Animals and cells

Human renal epithelial cells 293T, human hepatocellular carcinoma(HCC) cells HepG2, human gastric cancer cells MGC-803 and human lung cancer cells A549 were available from the American Type Culture Collection. 293 T, HepG2 and A549 cells were cultivated in DMEM (Gibco, USA) and MGC-803 cells were cultivated in 1640 medium (Gibco, USA) both emcompassing 10% fetal bovine serum (Hyclone, USA), penicillin (100 U/ml), along with streptomycin (100 μg/ml) at 37 °C with 5% CO_2_. Three patients with primary HCC were selected to obtain primary HCC cells that have been surgically removed(informed consent was obtained from all subjects). The study was approved by the institutional ethics committee of Hainan Medical University.

Female SPF-grade NOD/SCID mice with an age of 4–6 weeks were procured from Beijing Vital River Lab Animal Technology Co. Ltd (Beijing, China) and reared in an SPF environment. The Animal Ethics Committee of Hainan Medical University approved all protocols. all animal experiments comply with the ARRIVE guidelines and were performed in accordance with the National Institutes of Health guide for the care and use of Laboratory animals.

### LPS-Nb36 generation

The thin-film hydration method was employed for preparing maleimide-modified unloaded liposomes [30]. DDAB (Dimethyldioctadecylammonium), cholesterol, POPC (1-palmitoyl-2-oleoyl-sn-glycero-3-phosphocholine), PEG_2000_-DSPE (1,2-distearoyl-sn-glycerol-3-phosphoethanolamine-N-[amino(polyethylene glycol)-2000]), and MAL-PEG_2000_-DSPE were dissolved in chloroform with a molar ratio of 51.8:40:5:3:0.3 and added to a round-bottomed flask, followed by rotating evenly and sealing. Next, the flask was placed in the rotary evaporator (model: RE-52C, Yongguangming company, Beijing, China) for a 10-min rotation to form a layer of liposome at the bottom of the flask. After drying in a vacuum drying oven, 1 mL HEPES buffer was added, and ultrasound was carried out in a water bath ultrasound machine for 5 min to obtain a white suspension. The white suspension was placed into the extruder and subsequently filtered 20 times with 200 nm and 100 nm polycarbonate membranes and 21 times with 50 nm polycarbonate membranes, respectively, to obtain maleimide-modified unloaded liposomes, which were loaded into EP tubes and stored at 4 °C.

Nbs were connected to the surface of liposomes by the post-insertion method [31]. Nb36 (dithiothreitol treated) and MAL-PEG_2000_-DSPE were blended in a flask with a molar ratio of 1:10, followed by sealing with nitrogen and 24-h incubation at 4 °C. Finally, HEPES liquid dialysis was used to remove the free small molecules and obtain LPS-Nb36. LPS-CTLA-4 mAb was also prepared as described above.

The size, distribution range, together with the zeta potential of prepared LPS, LPS-CTLA-4 mAb and LPS-Nb36 were analyzed by dynamic laser particle size analyzer (NanoBrook 90plus PALS, Brookhaven, USA), and the morphology of liposomes was captured by transmission electron microscope (TEM, Jem-F 200, JEOL, Japan).

### Specific binding of LPS-Nb36

Since Nb36 was prepared with a His-tag, an anti His-tag monoclonal antibody (Abcam, UK, Cat No. ab213204) could be utilized to provide a fluorescent signal. After the co-incubation of LPS-Nb36 with activated CD8^+^ T cells, flow cytometry was implemented to test the His-tag expression in the CD8^+^ T cells that had been incubated with anti His-tag mAb. The above steps were then repeated, and the samples were stained with 4′, 6-diamidino-2-phenylindole (DAPI, Roche, Almere, The Netherlands). Images were acquired using a fluorescence microscopy.

In another part, these CD8^+^ T cells incubated with LPS-Nb36 were reincubated with anti-CTLA-4 mAb (Abcam, UK, Cat No. ab210384), and CTLA-4 expression was examined to reflect the specific binding of LPS-Nb36 to CD8^+^ T cells. Images were acquired using fluorescence microscope.

### CD8^+^ T cells and DCs generation

Blood cells for the study were harvested from healthy volunteers after signing informed consent. Firstly, human PBMCs were separated with density gradient centrifugation by using Human Lymphocyte Separation Medium (Solarbio, China), which was cultivated for 2 h in RPMI 1640 basic medium containing recombinant human IL-2 (100 U/mL; Sigma-Aldrich, USA). The adherent cells and non-adherent cells (mainly lymphocytes) were collected, respectively. Then, CD8^+^ T cells were further sorted out using Act SepTM CD3/CD28 Sorting activated magnetic beads (T&L Biotechnolog, China), and the sorted CD8^+^ T cells were cultivated and then activated in the same medium. The adhered cells were subjected to incubation in RPMI 1640 complete medium encompassing rhGM CSF (1000 U/mL; R&D, USA) and rhIL-4 (500 U/mL; Sigma-Aldrich, USA) for the induction of monocyte differentiation to DCs, and the non-adherent cells were regarded to be immature DCs. All human sample experiments were ratified by the Institutional Review Committee of Guangxi Medical University.

### Fusion of DC and tumor cells

Tumor cells (HepG2, A549, MGC-803 and primary HCC cells) at the logarithmic growth stage were collected, inactivated by 30 GyX irradiation, and mixed and cultured with PKH26 fluorescent dye (Sigma-Aldrich, USA). DCs cultured with CFSE fluorescent dye (Sigma-Aldrich, USA) were blended with tumor cells at 2:1 ratio, and the mixture was added into a 50 ml centrifuge tube. During this process, PEG (Sigma, USA) was slowly and gently added along the wall of the tube and was water-bathed for 5 min at 37 °C. The diluted collagen I (Sigma, USA) was then added and soaked in a 37 °C water bath for 30 min.

The cells, upon PBS washing, were suspended again. The fusion of tumor cells and DCs was viewed by fluorescence microscopy (Nikon ECLIPSE 80i, Nikon, Japan). To assess the DC maturation, the fusion cells were collected after 7 days of culture, and MHC II(Abcam, UK, Cat No. ab55152), CD80(Abcam, UK, Cat No. ab134120), and CD86(Abcam, UK, Cat No. ab239075) expression levels were tested by flow cytometry (Backman CytoFlex S). FlowJo v10.0 was utilized for data analysis. The fluorescence intensity of fusion cells was captured under a fluorescence microscope.

### Cell proliferation assay

For the purpose of addressing whether LPS-Nb36 could induce CD8^+^ T cell proliferation, individual groups of CD8^+^ T cells were pre-marked with PKH26 and subsequently co-cultivated for 5 days with DC/HepG2 fusion cells, DC/A549 fusion cells, or DC/MGC-803 fusion cells at a 10:1 ratio. These cells were cultivated with 50 μl/mL of Nb36, LPS-Nb36 (equimolar quantity of 50 μl/mL Nb), LPS (dose equivalent to LPS-Nb36) or an equimolar amount of anti-CTLA-4 mAb and LPS-CTLA-4 mAb. The fluorescence of PKH26 on CD8^+^ T cells was examined by the aforesaid flow cytometry.

### Cytotoxicity assay

For cytotoxicity assay, individual groups of CD8^+^ T cells were cultivated for 6 h at a ratio of 1:1, 5:1, 10:1, or 1:3 with PKH26-labeled target cells (HepG2, A549, MGC-803 and primary HCC cells). The above doses of Nb36, anti-CTLA-4 mAb, LPS, LPS-CTLA-4 mAb and LPS-Nb36 were added respectively. These target cells were then harvested and dyed with PI (Sigma-Aldrich, USA). Next, the ratios of PKH26^+^PI^+^ dead cells in each group were estimated with flow cytometry as a % of the specific lysis.

Moreover, CD8^+^ T cells in every group were subjected to 6-h cultivation with target cells at 20:1 ratio and dying with PE-anti CD107a antibodies (Biolegend, China, Cat No. 328607). The assay of the intracellular IFN-γ assays was implemented with a Cytofix/Cytoperm™ Fixation/Permeabilization Kit (BD Biosciences, USA). CD8^+^ T cells treated in the above steps were incubated with monensin (Beyotime, China) to inhibit surface protein transport, and cells were fixed with paraformaldehyde. The cells, with the raptured cell membranes, were stained with the anti-human IFN-γ mAb. The ratios of positive T cells were estimated by using flow cytometry.

### ELISA and ELISPOT assays

CD8^+^ T cells upon activation were co-cultivated for 24 h with HepG2, A549, or MGC-803 cells at a ratio of 1:1. IL-2, TNF-α, IFN-γ, and IL-10 levels in the cell supernatants were evaluated with ELISA with commercial specific ELISA kits (BD Biosciences, USA).

Meanwhile, the proportion of effector cells that specifically secreted IFN-γ was evaluated by ELISPOT assay. The activated CD8^+^ T cells were subjected to 24-h co-cultured with fusion cells of DC/HepG2, DC/A549, or DC/MGC-803 at 10:1 ratio in the ELISPOT plate. The above doses of Nb36, anti-CTLA-4 mAb, LPS, LPS-CTLA-4 mAb and LPS-Nb36 were added respectively. Afterward, the IFN-γ specific immune complex was processed by streptavidin-AP and visualized in the substrate solution (BCIP/NBT). A ImmunoSpot S6 Ultimate-V analyzer (Cellular Technology Limited) was employed to detect the number of spots.

### Modeling of xenograft tumors and in vivo therapy

Female inbred SCID mice (SPF, 16–18 g, 4-week-old) were injected subcutaneously with HepG2, A549, or MGC-803 cells (5 × 10^5^ cells/mouse) via the left armpit. The tumor volume, calculated with 0.5*ab*^2^ (a, largest diameter/b, perpendicular diameter) was recorded every 3 days with the assistance of vernier calipers. When tumors reached around 100 mm^3^, mice were randomized into several groups (5 mice/per group, use a random number table) and then injected with DC/tumor fusion vaccine-activated CD8^+^ T cells (1 × 10^7^ cells/per mouse) via the tail vein, and these cells were pre-incubated with LPS-Nb36, LPS-CTLA-4 mAb, Nb36, anti-CTLA-4 mAb and LPS, every 7 d for 4 times. The mice in the PBS and the CD8^+^ T cell control groups were injected with the vehicle PBS and naïve CD8^+^ T cells, separately. After the last treatment, mice were euthanized, with their tumors removed and retained for further assays. Implement open experiments in the animal experiment section.

### Immunohistology

Tissue samples were subjected to fixation in 10% neutral formalin, paraffin-embedding, and sectioning into 4-µm-thick pieces. To confirm the toxicity of the CAR T cells, we collected normal tissues from mice (brain, heart, liver, spleen, and lung). The tissue sections were collected and dyed with both hematoxylin and eosin (HE).

In order to investigate the tumor cell viability and the effector T cell infiltration in the tumor, the tumor tissues which had been treated with different groups of effector T cells and the tumor tissue sections (4 µm) were subjected to immuno-staining with anti human primary antibodies (Ki-67 and CD3) (Maixin Biotech, China). Afterward, the combined antibodies were first reacted with biotinylated secondary antibodies before testing with a Streptavidin-Peroxidase kit coupled with 3,3′-Diaminobenzidine (DAB, Maixin Biotech, China). The latter contained secondary biotinylated antibodies and streptavidin/horseradish peroxidase complex. The immunofluorescence method was employed to determine apoptosis (TUNEL, FITC, in situ Cell Death Detection Kit, Roche, Switzerland) and the microvascular densities (CD34, BD Biosciences, USA) in the paraffin sections of the tumor tissues following the supplier’s instructions. Images were captured under a fluorescence microscope.

### In vivo persistence and antitumor efficacy of CD8^+^ T cells in DC/tumor fusion + LPS-Nb36 treatment

After receiving DC/tumor fusion + LPS-Nb36 treatment, five modeled mice in each group were sacrificed on days 7, 14, and 21. The spleen, peripheral blood, along with tumor tissues of each mouse, was collected, and the latter two were chipped into small pieces and susbsequently homogenized. After incubation with the anti-human CD3 mAb for fluorescence staining, the ratio of CD3 level was appraised by flow cytometry. The determination of IFN-γ and IL-2 serum levels was achieved using ELISA on day 21.

### Statistical methods

GraphPad Prism 6.02 software was implemented for statistical analysis. Data were analyzed with students’ t-tests and an analysis of variance (ANOVA). The different survival curves of animals between groups were plotted using the Kaplan-Meier analysis (log-rank test). Statistical significance was defined as a *p*-value below 0.05. All data were depicted as means ± standard deviation (SD).

## Results

### Characterization and specific binding of LPS‑Nb36

The characteristics of LPS‑Nb36 were analyzed after preparation. TEM was employed to observe the shape characteristics, dispersion, and size of LPS-Nb36. Our data showed that the distribution of LPS-Nb36 was almost monodisperse, and the size was relatively uniform, spherical, or elliptical spherical (Fig. S2A). The sizes of LPS-Nb36, LPS-CTLA-4 mAb and LPS shown in the picture were smaller than those determined by the dynamic laser particle size analyzer. The reason may be that the liposomes observed under TEM are in a dry state, and those measured by the particle size analyzer are in an aqueous environment in which the liposomes have a hydrated membrane to increase their particle size [32]. The mean particle size of LPS-Nb36 was approximately 110 nm (Fig. S2C and Table 1) with a low PDI (Table 1), suggesting that the distribution of these particles is very uniform, and the ZETA potential results also suggest the high stability of LPS-Nb36 (Fig. S2B).

**Table 1:** The hydrodynamic size and polydispersity index(PDI) of LPS-Nb36.

	Hydrodynamic size (nm)	PDI
LPS	112.45 ± 0.80	0.197 ± 0.003
LPS-Nb36	118.18 ± 2.64	0.201 ± 0.002

We further examined whether LPS-Nb36 could specifically bind to the activated T cells. Since the Nb36 has a His-tag at the time of preparation, an anti His-tag mAb could be utilized to provide a fluorescent signal. When LPS-Nb36 was incubated with activated CD8^+^ T cells, the His-tag binding efficiency reached 38.4%, suggesting that these Nb-modified liposomes have a strong binding ability to the CTLA-4 antigen (Fig. S3A). Furthermore, fluorescence microscopy was performed to visualize the target cells binding of LPS-Nb36. The high fluorescence intensity in the activated CD8^+^ T cells indicated that a high amount of LPS-Nb36 were specifically bound to CTLA-4 on the cytomembrane (Fig. S3B).

In contrast, we continued to detect the binding efficiency of CTLA-4 in these CD8^+^ T cells incubated with LPS-Nb36 using a CTLA-4 mAb and found that the binding efficiency of CTLA-4 in these T cells was notably reduced relative to the un-incubated T cells. Collectively, the CTLA-4 antigen on T cells actually binds specifically to LPS-Nb36.

### LPS‑Nb36 has no toxicity in cellular and animal assays

Subsequently, the CCK-8 assay and HE staining were employed to appraise the potential toxicity of LPS‑Nb36 in cellular and animal assays, separately. The viability of CD8^+^ T cells and Jurkat cells was not markedly altered upon 24 h- or 48 h-treatment with LPS‑Nb36 at concentrations from 100 to 1000 nM (Fig. S4A). Likely, no signs of toxicity were witnessed in the kidney, lung, spleen, liver, and heart tissues of mice with an intravenous injection of LPS‑Nb36 at the therapeutic dose (Fig. S4B). These findings confirm the safety of LPS‑Nb36 in cellular and animal assays.

### Characterization of DC/tumor fusion cells

**Fig. 2:**
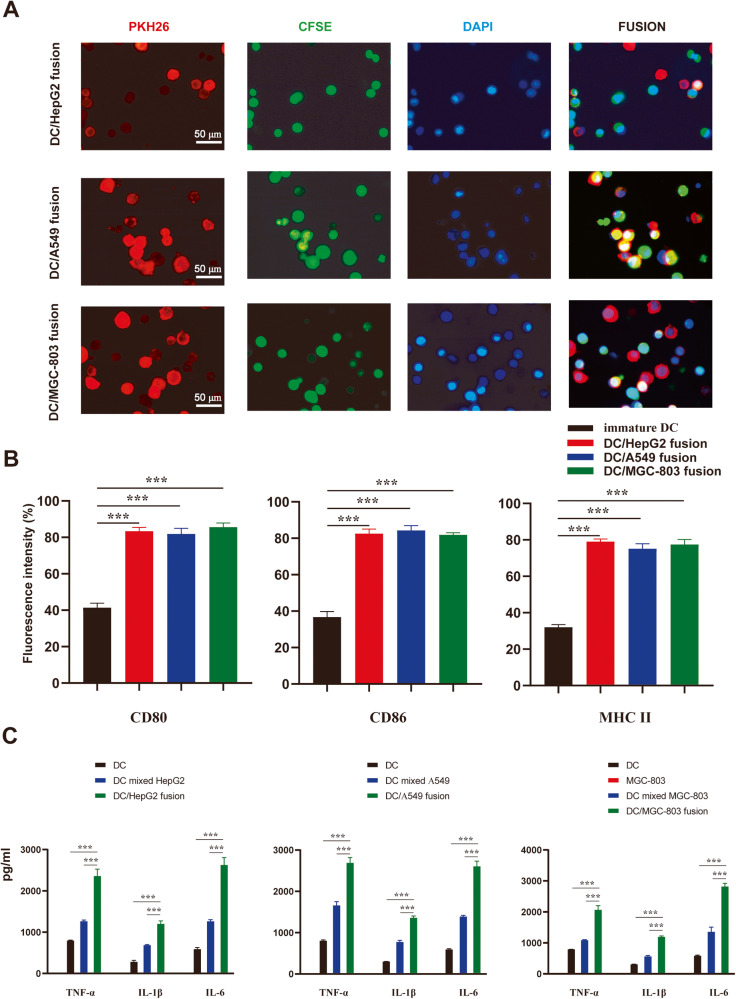
Fusion of DCs and tumor cells. **A** DCs and HepG2, A549 or MGC-803 cells were co-cultured in the presence of PEG 2000, respectively. DCs were stained with PKH26 (red), tumor cells were stained with CFSE (green),and nuclei were stained with DAPI (blue). Scale bar = 50 μm. **B** DC/tumor fusion cells expressed high levels of costimulatory CD80, CD86 and MHC II molecules. *n* = 3, ***P < 0.001. **C** Fusing DCs with corresponding tumor cells enhanced expression of DC-specific cell surface molecules (TNF-α, IL-1β, and IL-6). *n* = 3, ***P < 0.001.

The tumor cells stained with CFSE and DCs stained with PKH26 after co-cultured for 7 days in the presence of PEG, our resulting DC/tumor fusion cells showed both kinds of fluorescence and high expression marker associated with maturity (Fig. 2A, B). Furthermore, these DC/tumor fusion cells produced higher levels of TNF-α, IL-1β and IL-6 than DCs cultured alone or simply mixed with tumor cells (Fig. 2C). These results confirm that DC/tumor fusion activates DCs by promoting their maturation.

### LPS‑Nb36 advances CD8^+^ T cell proliferation and activation in DC/tumor fusion + LPS-Nb36 treatment

**Fig. 3:**
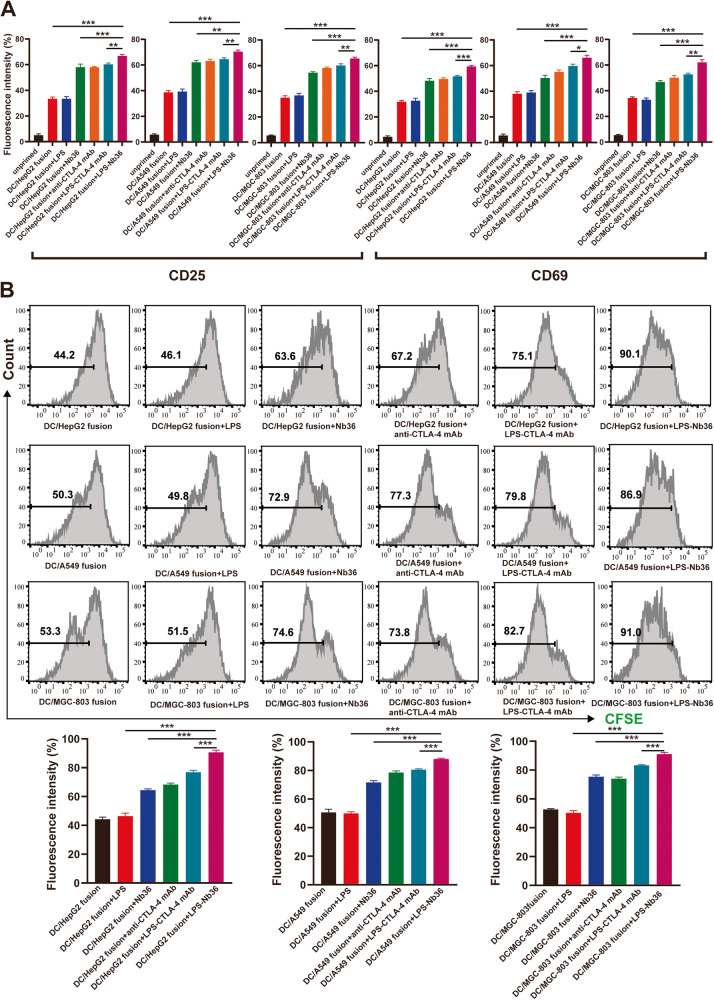
LPS-Nb36 enhances activation and proliferation of CD8^+^ T cells in DC/tumor fusion + LPS-Nb36 treatment. **A** The CD8^+^ T cells were stained with CFSE and co-culture with HepG2, A549 or MGC-803 cells, respectively, with LPS-Nb36, Nb36 or anti-CTLA-4 mAb for 5 days. CD8^+^ T cells alone as the blank control group(unprimed). The effector cells were stained with PE-conjugated anti-CD25 or anti-CD69 mAb and analyzed by flow cytometry for the activation. *n* = 3, **P < 0.01, ***P < 0.001. **B** The fluorescent intensity of CFSE in the effector cells was directly analyzed by flow cytometry for the proliferation. *n* = 3, **P < 0.01, ***P < 0.001.

The sorted out autologous T cells expressed CD8 more than 80 percent (Fig. S1). LPS‑Nb36 is capable of enhancing DC/tumor fusion vaccine-induced autologous CD8^+^ T cell proliferation and activation. Our data indicated that LPS‑Nb36 together with DC/HepG2 fusion vaccine, DC/A549 fusion vaccine, or DC/MGC-803 fusion vaccine significantly upregulated CD25 and CD69 expression on the CD8^+^ T cells’ surface (*p* < 0.001; Fig. 3A). In the meantime, in co-culture with these three DC-tumor fusion vaccines, LPS‑Nb36 more significantly advanced these CD8^+^ T cell proliferation (*p* < 0.001) (Fig. 3B). To conclude, LPS‑Nb36 in a combination with DC/tumor fusion vaccine can boost CD8^+^ T cell proliferation and activation. Moreover, the use of anti-CTLA-4 Nb-modified liposomes as a T cell CTLA-4 blocker has a higher efficiency than Nb in this treatment.

### LPS‑Nb36 strengthens the secretion of inflammatory cytokines and cytotoxicity of CD8^+^ T cells in DC/tumor fusion + LPS-Nb36 treatment

**Fig. 4:**
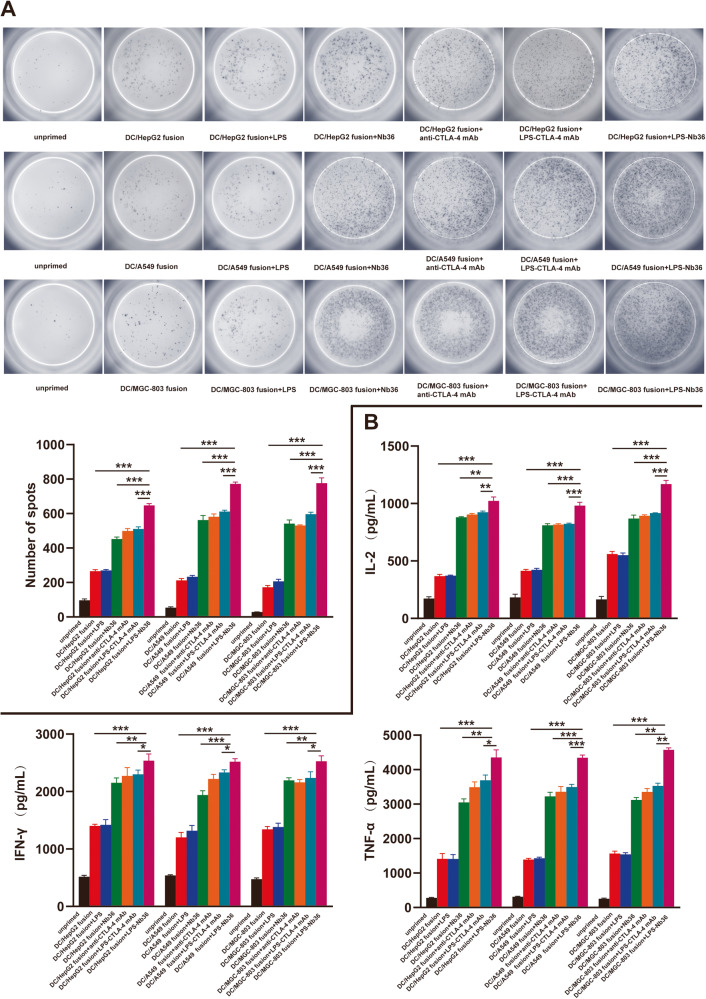
LPS-Nb36 enhances inflammatory cytokine secretion of CD8^+^ T cells in DC/tumor fusion + LPS-Nb36 treatment. The sorted human CD8^+^ T cells were cultured and activated by corresponding DC/tumor fusion vaccine alone or in the presence of LPS-Nb36, Nb36, LPS-CTLA-4 mAb or anti-CTLA-4 mAb, then stimulated with the same number of irradiated HepG2, A549 and MGC-803 cells for 24 h in vitro. CD8^+^ T cells cultured without DC/tumor fusion vaccine as the unprimed group. **A** The secretion of IFN-γ in the activated CD8^+^ T cells of different groups against the stimulations of corresponding tumor cells was detected by ELISPOT assay to indicate the significant involvement of LPS-Nb36 with the increased amount of IFN-γ-producing CD8^+^ T cells inducing by DC/tumor-FC vaccines. Representative image of ELISPOT plate readout analyzing the frequency of IFN-γ-secreted CD8^+^ T cells are shown histograms represent data of the triplicates for 3 × 10^5^ cells from three independent ELISPOT assays, and shown as bars of means + SD. ***P < 0.001. **B** After co-culture with effector cells and target cells, supernatants were separated and analyzed for secretion of IFN-γ, TNF-a and measuring by the ELISA kits. Results indicated the increased production of above three cytokines from the tumor cell-reactive CD8^+^ T cells mediated by LPS-Nb36.Bar graphs showmean ofcytokine concentration + SD. *n* = 3, **P < 0.01, ***P < 0.001.

ELISPOT results elucidated that an elevated number of IFN-γ secreting spot forming was witnessed in the DC/tumor fusion+LPS‑Nb36 groups in contrast to those in the DC/tumor fusion, DC/tumor fusion+anti-CTLA-4 mAb, DC/tumor fusion+LPS-CTLA-4 mAb and DC/tumor fusion+Nb36 groups after being challenged with corresponding target cells (Fig. 4A). Thus, these data imply that LPS‑Nb36 enhanced the genration of pro-inflammatory mediators by CD8^+^ T cells during stimulation by DC/tumor fusion vaccine, which would advance local inflammatory response and attack on tumor cells.

To address the secretion of cytokines in activated CD8^+^ T cells, we performed ELISA to examine the supernatant of these T cells in DC/tumor fusion+LPS-Nb36 treatment. Our data demonstrated LPS‑Nb36 treatment coupled with irradiated DC/HepG2, DC/A549, or DC/MGC-803 fusion cells contributed to an enhanced release of inflammatory mediators (TNF-α, IFN-γ, and IL-2) in contrast to the DC/tumor fusion groups (*p* < 0.001) (Fig. 4B). These cytokine levels also higher than those in response to treatment of Nb36 with the corresponding DC/tumor fusion cells. Notably, no difference was detected in IL-10 level among all the groups (*p* > 0.05) (Fig. S5).

**Fig. 5:**
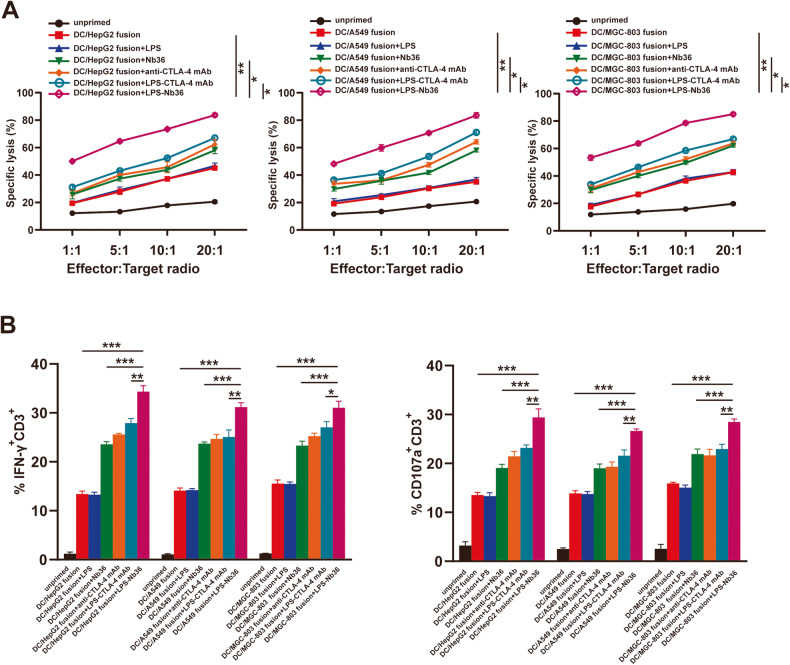
LPS-Nb36 enhances cytotoxicity of CD8^+^ T cells activated by DC/tumor fusion vaccine. **A** The CD8^+^ T cells were co-incubated with PKH26-prestained HepG2, A549 or MGC-803 cells with DC/tumor fusion vaccine at *E*/*T* ratio 1:1,5:1,10:1 or 20:1, with LPS, LPS-Nb36, Nb36, anti-CTLA-4 mAb or LPS-CTLA-4 mAb for 6 h. propidium iodide(Pl)was used for lysed cell staining. The ratios of PHK26^+^PI^+^cell were measured by flow cytometry. *n* = 3, *P < 0.05, **P < 0.01. **B** After co-culturing with CAFs at a 20:1 ratio for 6 h, the IFN-γ(intracellular) and CD107a expression of effector cells were measured by flow cytometry. *n* = 3, ***P < 0.001.

CD8^+^ T cells could eliminate more corresponding target cells in response to the treatment of LPS‑Nb36 coupled with the DC/tumor fusion vaccine. These CD8^+^ T cells showed significantly enhanced cytotoxicity with an increasing efficiency-target ratio. In contrast, their cytotoxic impact on the target(HepG2, A549, MGC-803 and primary HCC cells) was also remarkably higher than in the DC/tumor fusion and DC/tumor fusion+Nb36 groups (Fig. 5A and Fig. S6A, B), further demonstrating that the enhancement effect of LPS‑Nb36 is better than that of Nb36.

To further demonstrate the high cytotoxicity of the CD8^+^ T cells upon treatment of LPS-Nb36 and DC/tumor fusion vaccine against corresponding target cells, we measured the IFN-γ intracellular expression by flow cytometry and the mobilization of CD107a (representing the cytotoxic phenotype) across the membrane of these CD8^+^ T cells, The findings unveiled an upregulated IFN-γ and CD107a expression in DC/tumor fusion + LPS-Nb36 groups (Fig. 5B), which is consistent with the outcomes of the cytotoxicity assay.

### DC/tumor fusion + LPS-Nb36 treatment leads to a higher CD8^+^ T cell effector function in xenografted mice

**Fig. 6:**
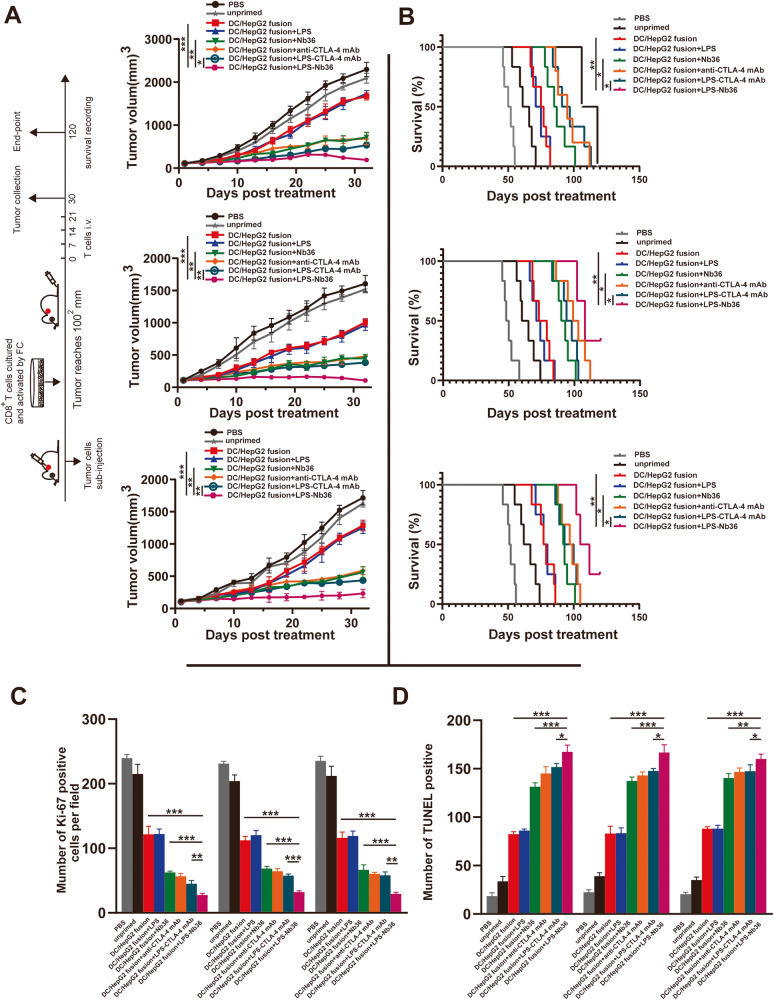
DC/tumor fusion + LPS-Nb36 treatment lead to a higher CD8^+^ T cell effector function in xenograft mice. **A** The tumor size was measured every 3 days. *n* = 5, **P < 0.01, ***P < 0.001. **B** Kaplan–Meier survival curve of each group. *n* = 5, *P < 0.05, **P < 0.01. **C** Ki-67 detection by immunohistochemical stain. *n* = 5, ***P < 0.001. **D** The number of apoptosis cells detection by TUNEL. *n* = 5, **P < 0.01, ***P < 0.001.

To explore the mechanism of tumor growth inhibition in mice following treatment with DC/tumor fusion+LPS-Nb36, we established xenograft models with the corresponding tumor cell line (HepG2, A549, and MGC-803) to observe the influence of this combined treatment on the xenograft tumor growth in mice. A comparison was made on the tumor growth curves and survival curves for these three models which had been treated with CD8^+^ T cells (activated by DC/tumor fusion, DC/tumor fusion vaccine+LPS, DC/tumor fusion vaccine+anti-CTLA-4 mAb, DC/tumor fusion vaccine+LPS-CTLA-4 mAb as well as DC/tumor fusion vaccine+LPS‑Nb36), naïve T cells, and PBS, respectively. Our data indicated that CD8^+^ T cells in response to the activation of the DC/tumor fusion vaccine and LPS‑Nb36 retarded tumor growth and strengthened survival of xenografted mice, which performed better treatment results than other groups (Fig. 6A).

The mechanism of the xenograft model, following DC/tumor fusion+LPS-Nb36 treatment, was confirmed by the detection of Ki-67 and apoptosis in the tumor through immunohistochemical staining or immunofluorescence staining. Our results indicated a lower number of Ki-67-positive cells in the DC/tumor fusion +LPS‑Nb36 group in comparison to the DC/tumor fusion and DC/tumor fusion + Nb36 groups. This result correlated with the significant apoptosis detected by the TUNEL assay. Collectively, our data indicate that adoptive CD8^+^ T cells upon the activation of DC/tumor fusion vaccine and LPS-Nb36 restrict the implanted hepatoma tumors progression in mice by impeding tumor cell proliferation and boosting its apoptosis (Fig. 6B).

### Long-term antigen-dependent persistence of CD8^+^ T cells with the activation of DC/tumor fusion vaccine and LPS-Nb36 in vivo

**Fig. 7:**
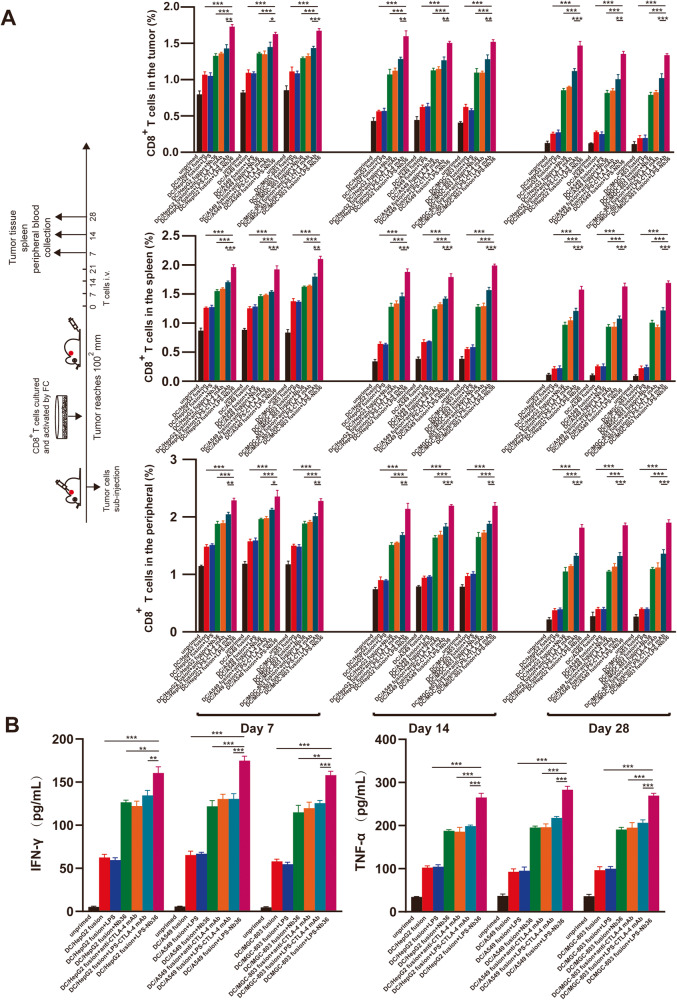
Long-term antigen-dependent persistence of CD8^+^ T cells activated by DC/tumor fusion vaccine + LPS-Nb36 in vivo. **A** The percentage of human CD8^+^ T cells in a tumor, spleen, and blood of tumor-bearing mice that were adoptively transferred with effector T cells in each groups were measured by flow cytometry on days 7, 14, and 28. *n* = 3, *** *P* < 0.001. **B** Human IFN-γ and TNF-α levels in tumor-bearing mice serum were detected by ELISA after adoptively transferring with effector T cells in each grouups. *n* = 3, **P < 0.01, ***P < 0.001.

Next, the CD8^+^ T cell persistence in each group was evaluated in vivo on days 7, 14, and 28, after a full course of treatment. After euthanasia, different tissues, including the tumor, blood, as well as spleen, were obtained from mice for human CD8 staining. The data from flow cytometry demonstrated that the CD8^+^ T cells activated by DC/tumor fusion vaccine + LPS-Nb36 persisted throughout the experiment. Upon 28-day administration, these CD8^+^ T cells were slightly lower in the tumor, blood, as well as spleen, but still displayed persistent antitumor activity (Fig. 7A). In this part of the experiment, we also performed cytokine testing on the blood from mice that had been collected on day 28. We found that human IFN-γ and TNF-α serum levels were far higher in the DC/tumor fusion +LPS-Nb36 group versus those in the others (Fig. 7B). These findings imply that CD8^+^ T cells might survive longer in mice and continuously promote local inflammatory response and attack tumor cells.

## Discussion

In the last decade, two types of immunotherapy, namely the application of immune checkpoint inhibitors to advance natural antitumor activity [33] and the utilization of specific antitumor immune cells through adoptive cell therapy (ACT) [34] have been regarded to be particularly effective in cancer therapy. Both of these two immunotherapies have undergone tremendous technological updates as independent research and development routes and have been accompanied by the launch of corresponding drugs. However, from the current perspective, tumor immunotherapy combined with multiple modalities is becoming the mainstream of research and development.

Indeed, the clinical application of ACT has achieved encouraging results in tumor therapy. Nevertheless, many challenges remain to be addressed to overcome the effectiveness, security, and economic barriers. Activated CD8^+^ T cells, known as CTLs, can induce tumor cell apoptosis by secreting perforin and granase, and kill target cells through the generation of cytokines (IFN-γ and TNF-α) in an indirect manner. When the tumor occurs, the CTLs produced against the tumor antigen are called tumor-specific T cells, which are usually difficult to obtain in large quantities through autologous sorting and are mainly induced in vitro. However, their activation requires APCs, especially DCs, to transmit corresponding antigenic signals to recognize tumor cells [35]. Early studies have also recognized that adoptive therapy of unactivated CD8^+^ T cells fail to differentiate into tumor-specific T cells and achieve high antitumor efficacy. Therefore, a variety of means have been created to deliver TAAs to DCs as cancer vaccines [36]. Approaches for introducing tumor antigens into DCs include transfecting with RNA or DNA, pulsing with peptides or proteins, as well as the utilization of fusion between tumor cells and DCs [7, 37]. Nevertheless, the specific CTLs induced by DCs/tumor vaccines are still far from satisfactory especially for in vivo applications. The tumor-specific CTLs’ induction and activation are hindered by many factors in vivo, the most important of which is the negative regulation from immune checkpoints. According to several DC/tumor fusion vaccines constructed in our previous studies [38, 39], the survival time of effector T cells in mice did not meet expectations, and they could not continuously hinder tumor growth and recurrence.

In order to enhance CD8^+^ T cell proliferation and viability, we interrupted their CTLA-4/B7 pathway and used Nb36 (a special anti-CTLA-4 Nb) as an immune checkpoint blocker. Nb36 has shown good antigen-binding ability and stability in our previous studies [26, 27]. Due to its very small molecular weight and stability, Nb is more suitable for modification to form enrichment on the vector than mAb, thus enhancing its antigen recognition and binding ability [40]. Based on this, we decided to construct LPS-Nb36 using the currently well-established liposome synthesis/modification techniques. In addition, considering that the follow-up research may be applied to humans, the modification of Nb36 to liposome can theoretically prolong his half-life in vivo. As shown in our data, these synthesized liposomes have uniform particle size and dispersion with no significant change in the characterization of Nb36 after modification. Nb36 can specifically bind to CTLA-4 antigen in vitro, and its binding ability seems to be no less than the anti-CTLA-4 mAb. The constructed LPS-Nb36 was utilized as an efficient CTLA-4 blocker to diminish the negative influence of the CTLA-4/B7 axis on the CD8^+^ T cell activation. The DC/tumor fusion vaccine was simultaneously activate CD8^+^ T cells and transmit tumor antigens which induce the tumor-targeting ability, survival, as well as cytotoxicity of CTLs. The findings of our paper confirm a notable synergistic effect of anti-CTLA-4 Nb-modified liposomes-based CTLA-4 checkpoint blockade and CD8^+^ T cells activated by the DC/tumor fusion vaccine. The circulating adoptive CD8^+^ T cells activated by this treatment possess a high tumor-targeting ability to gather into the tumor site, thereby boosting tumor cell apoptosis and pro-inflammatory reaction, Therefore, anti-CTLA-4 Nb-modified liposomes-mediated CTLA-4 blockade has great potential for clinical application by acting as a novel strategy for interrupting T cell depletion from immune checkpoints that can be easily used for adoptive therapy in activated T cells.

The purpose of cancer adoptive therapy is to explore enhancing the capability of immune cells to selectively target and dispel tumor cells while minimizing damage to normal tissues. However, the lack of specificity for the heterologous reaction results in treatment-related significant toxicity and mortality. By processing newly synthesized and internalized antigens to optimize antigen presentation along class I and class II pathways, DC vaccines are expected to address the problem of effector T cells targeting tumor cells [41], and preclinical studies have shown that DC vaccines can effectively stimulate antitumor immunity [42]. In this present study, the DC vaccine was prepared by the fusion method. Theoretically, DCs infused with tumor cells would lead to known and unknown broad-spectrum tumor antigens in the context of an effective immunostimulatory mechanism for DCs [43]. For improving the CD8^+^ T cell antigenic specificity and cytotoxicity to tumor cells, we designed 3 DC/tumor vaccines through fusing immature DCs with three common tumor cells (HepG2, A549, and MGC-803). The maturity of DC/tumor fusion cells is usually determined by costimulatory molecules (e.g. CD80, CD86, as well as NHC-II) expression levels. These three types of DC/tumor fusion vaccines presented elevated expression levels of these indicators than those of mature and immature DCs, suggesting that the fusion of DCs with tumor cells promoted its own maturation and improved antigen presentation capacity. We further detected the cytokine secretion by these DC/tumor fusion cells, with the results fully confirming the successful construction of DC/HepG2, DC/A549, and DC/MGC-803 fusion cells.

The CTLA-4/B7 signal pathway transmits negative signals to effector T cells to further weaken their activation. As reported, the impacts of the DC/tumor fusion vaccine are coupled with the impacts from the mAb targeting CTLA-4 [44]. Indeed, several antibodies specific to PD-L1 or CTLA-4 PD-1 have been recommended for the therapy of diverse cancers [45]. Nevertheless, the monoclonal antibody drugs with large molecular weight result in the depletion of activated lymphocytes to restrict their permeation and concentration in solid tumors, thereby causing poor effect of tumor treatment [46]. Furthermore, the high costs hinder its widespread application. Nbs have the inherent characteristics of much smaller molecular weight and the absence of Fc domain, which can be widely used in the treatment and detection of tumors. Therefore, Nbs can have great development potential as alternatives to monoclonal antibodies. In our previous study [47], we attempted to activate CIK cells with Nb36 and observed a certain degree of enhanced cell activity, which enhanced the killing power of CIK cells. However, this effect is neither specific nor long-lasting. As expected, in this work, the CD8^+^ T cells activated by DC/tumor fusion vaccine in a combination of LPS-Nb36 could rapidly transmit T cell activation signals after encountering tumor antigens, as demonstrated by upregulated CD25 and CD69 expression, and enhanced secretion of cytokines (TNF-α, IFN-γ, and IL-2). In addition, it is worth mentioning that we also constructed an DC/primary HCC cells vaccine in the supplementary experiment. Our data confirmed that DC/tumor fusion vaccine+LPS-Nb36 treatment also had a very significant cytotoxicity on primary tumor cells. To conclude, LPS-Nb36 has a stronger antigen-binding ability than Nb36 and can more efficiently block the CTLA-4 signal pathway of CTLs activated by the DC/tumor fusion vaccine.

To substantiate the CD8^+^ T cell activity activated by DC/tumor fusion vaccine+LPS-Nb36 treatment in damaged tumor cells in vivo, we established HepG2, A549, and MGC-803 subcutaneous xenograft mice models. Our data substantiated that the utilization of these CD8^+^ T cells retarded tumor growth and strengthened the survival rates of all three noduliferous mice. Studies on the mechanism of killing tumors by these CD8^+^ T cells disclosed a significant decrease in the proliferation (Ki-67) and an elevation in apoptotic cells of tumor cells in mice, which was consistent with the specific killing of CTLs on tumor cells. More importantly, we tested the survival of these T cells in mice. The restriction of adoptive T cells combined with a highly invasive solid tumor may be responsible for tumor recurrence. A long-lasting remission for solid tumors seems to be dependent on the long-term persistence of infused effector T cells. Our data demonstrated that the human CD8^+^ T cells with activation of DC/tumor fusion vaccine+LPS-Nb36 persisted for more than 4 weeks and were longer in tumor-bearing mice than in other groups of T cells, whether these cells were in the peripheral blood, spleen, or within the tumor itself. Persistent effector T cells maintain a more durable antitumor effect, and the IFN-γ and TNF-α levels in mouse serum are also observed to remain at high levels.

In summary, we have developed a novel anti-CTLA-4 Nb-modified liposome. Our findings underline that blocking the CTLA-4/B7 signal pathway on CD8^+^ T cells is not limited to the application of the anti-CTLA-4 mAb, instead, liposomes with modified or even smaller antigen receptors, such as LPS-Nb36, can be used. Compared with the application of Nb alone, this liposome complex enriched with Nb greatly enhanced its antigen recognition and binding ability. The specific binding of LPS-Nb36 and CTLA-4 greatly restricts the CTLA-4/B7 pathway. As a result, more costimulatory signals (CD28/B7) are produced, enabling T cells to overcome the negative effects and obtain stronger activation and proliferative ability.

Furthermore, we offered a preclinical experience for the treatment potential of LPS-Nb36. The CD8^+^ T cells activated by DC/tumor fusion vaccine in a combination with LPS-Nb36 can selectively engulf corresponding tumor cells, which appear to be a safe approach. This optimally-designed dual activation therapy might be beneficial for enhancing CD8^+^ T cell proliferation, cytotoxicity, as well as survival. Therefore, it is capable of improving existing immunotherapeutic regimens for malignancies. Furthermore, we hypothesize that this anti-CTLA-4 Nb-modified liposome has the potential to be directly applied or used in the construction of in vivo therapeutic drug delivery systems based on its toxicity verification, which will be the focus of our next study.

## Supplementary information

aj-checklist

Supplementary Figure legends

Supplementary Figure s1

Supplementary Figure s2

Supplementary Figure s3

Supplementary Figure s4

Supplementary Figure s5

Supplementary Figure s6

Supplementary table1

## Data Availability

The datasets generated and/or analyzed during the current study are available from the corresponding author upon reasonable request.
