# Supplementary material for: A novel CTLA-4 blocking strategy based on nanobody enhances the activity of dendritic cell vaccine-stimulated antitumor cytotoxic T lymphocytes
Source: Cell Death Dis. 2023 Jul 7;14(7):406. doi: 10.1038/s41419-023-05914-w (PMC10328924; doi:10.1038/s41419-023-05914-w)
Supplement: Supplementary file 2 — Supplementary Figure legends [file 41419_2023_5914_MOESM2_ESM.docx]

**Supplementary Figure S1. Purification and characterization of CD8^+^ T cells.** CD8 and CD3 markers were used to identify CD8^+^ T cells clusters before and after sorting(CD3/CD28 Sorting activated magnetic beads) by flow cytometry. *n*=3.

**Supplementary Figure S2. Generation and characterization of LPS-Nb36.** (A) TEM image for LPS-Nb36. 200× 100× (B) The Zeta Potential distribution of LPS-Nb36. *n*=3. (C) The size distribution of LPS-Nb36.

**Supplementary Figure S3. In vitro binding of LPS-Nb36 to CTLA-4-positive**

**cells. (A) To test the CTLA-4 binding specificity of LPS-Nb36, flow cytometry was used to detect the expression of the His-tag in the CD8^+^ T cells that had been incubated with anti His-tag mAb. Meanwhile, these CD8^+^ T cells incubated with LPS-Nb36 were again stained with anti CTLA-4 mab, and their CTLA-4 expression was detected by flow cytometry. (B) Fluorescence micrographs of** **activated CD8^+^ T cells and 293T cells after incubation with LPS-Nb36 and** anti-His tag mAb**.** activated CD8^+^ T cells and 293T cells were resuspended in PBS and incubated with LPS-Nb36 for 30 minutes, they were subsequently incubated with anti-His tag mAb at 4℃ for 30 minutes, stained with DAPI. Images were acquired using fluorescence microscope.

**Supplementary Figure S4. Toxicity assessment for LPS-Nb36.** (A)Viability of T cells and jurkat cells were determined using CCK8 assays after 24 h treatment with LPS-Nb36 and LPS. (B) Healthy mice were injected with LPS-Nb36 or PBS. Sections from primary organs were stained with hematoxylin-eosin and images were captured using confocal microscopy. Magnification, x400.

**Supplementary Figure S5**. **LPS-Nb36 enhances inflammatory cytokine secretion of CD8^+^ T cells in DC/tumor fusion+LPS-Nb36 treatment.** The sorted human CD8^+^ T cells were cultured and activated by coresponding DC/tumor fusion vaccine alone or in the presence of LPS, LPS-Nb36, Nb36, anti CTLA-4 mAb or LPS-CTLA-4 mAb , then stimulated with the same number of irradiated HepG2, A549 and MGC-803 cells for 24 h in vitro. CD8^+^ T cells cultured without DC/tumor fusion vaccine as the unprimed group. After co-culture with effector cells and target cells, supernatants were separated and analyzed for secretion of IL-10 by the ELISA kits. Results indicated the increased production of above three cytokines from the tumor cell-reactive CD8^+^T cells mediated by LPS-Nb36. Bar graphs showmean ofcytokine concentration + S.D. *n*=3, ***P* < 0.01, ****P* < 0.001.

**Supplementary Figure S6. LPS-Nb36 enhances cytotoxicity of CD8^+^ T cells activated by DC/tumor fusion vaccine against primary hepatocellular carcinoma(HCC) cells.** Three patients with primary hepatocellular carcinoma were selected to obtain tumor specimens that have been surgically removed. The tumor tissue with the size of 1.0 cm^3^ was cut under aseptic conditions, and the fibrous tissue in the tumor tissue was removed and put into a container containing RPMI 1640 culture medium, and cut into small pieces. After processing with 0.25% trypsin digestion solution, filter with nylon mesh to obtain primary HCC cells. (A) Fusion of DCs and primary HCC cells. DCs and primary HCC cells were co-cultured in the presence of PEG 2000. DCs were stained with PKH26 (red), primary HCC cells were stained with CFSE (green),and nuclei were stained with DAPI (blue). Scale bar= 50 μm. (B) The CD8^+^ T cells were co-incubated with PKH26-prestained primary HCC cells with DC/tumor fusion vaccine at E/T ratio 1:1,5:1,10:1 or 20:1, with LPS, LPS-Nb36, Nb36, anti CTLA-4 mAb or LPS-CTLA-4 mAb for 6 h. propidium iodide(Pl)was used for lysed cell staining. The ratios of PHK26^+^PI^+^cell were measured by flow cytometry. *n*=3, **P* < 0.05, ***P* < 0.01.
