# Supplementary figures and images for: A novel CTLA-4 blocking strategy based on nanobody enhances the activity of dendritic cell vaccine-stimulated antitumor cytotoxic T lymphocytes
Source: Cell Death Dis. 2023 Jul 7;14(7):406. doi: 10.1038/s41419-023-05914-w (PMC10328924; doi:10.1038/s41419-023-05914-w)

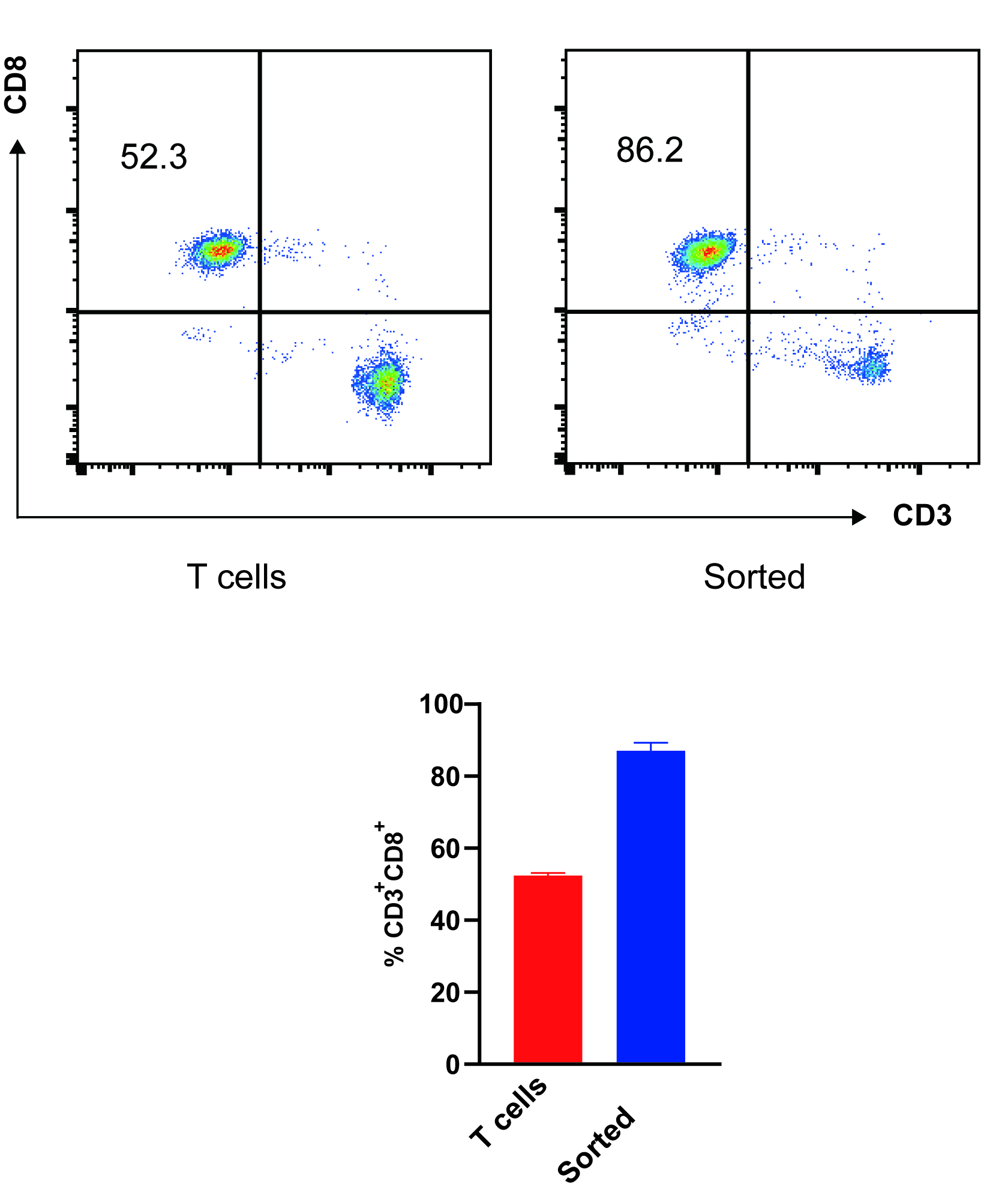

Supplement: Supplementary file 3 — Supplementary Figure s1 [file 41419_2023_5914_MOESM3_ESM.tif]

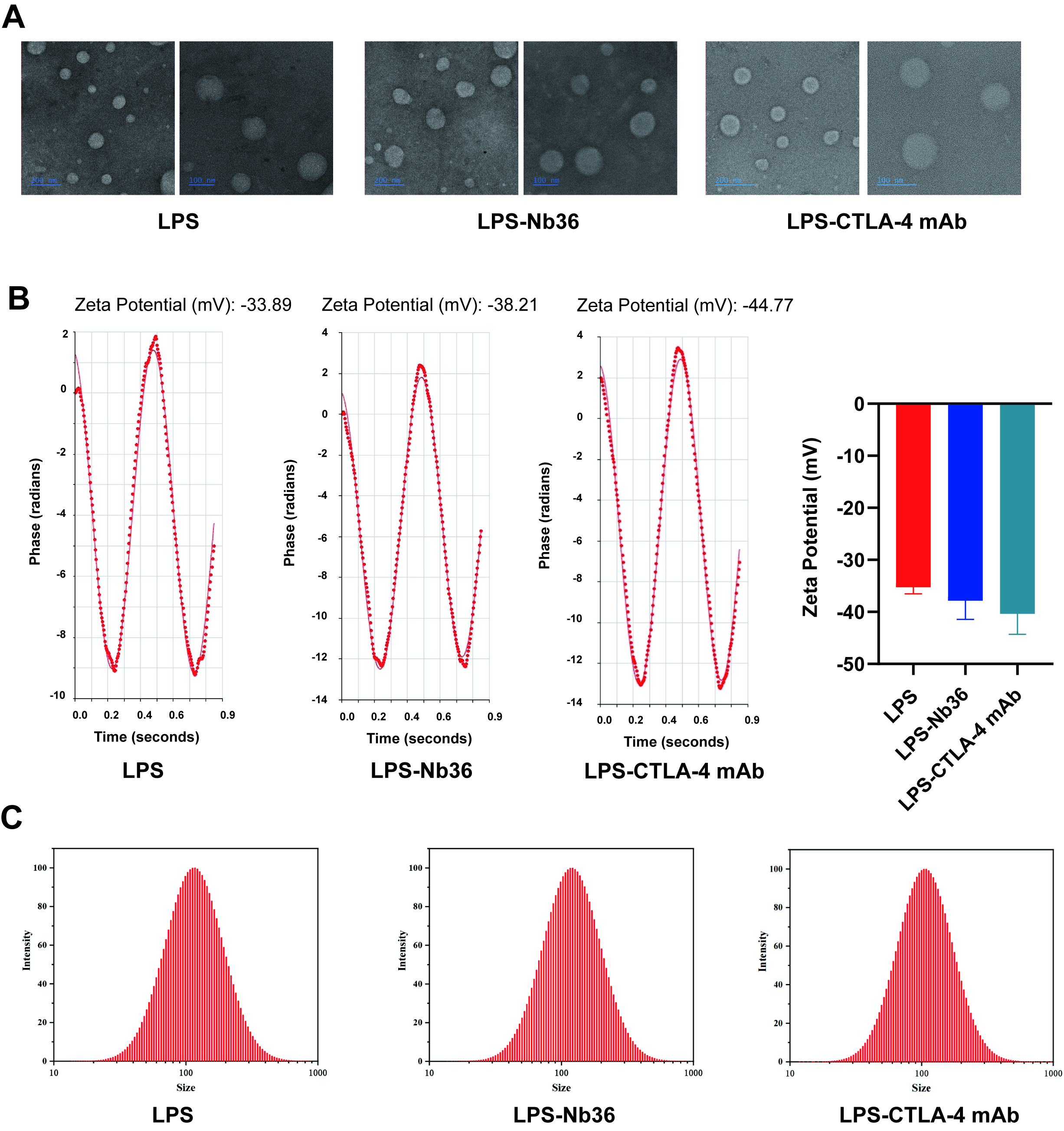

Supplement: Supplementary file 4 — Supplementary Figure s2 [file 41419_2023_5914_MOESM4_ESM.tif]

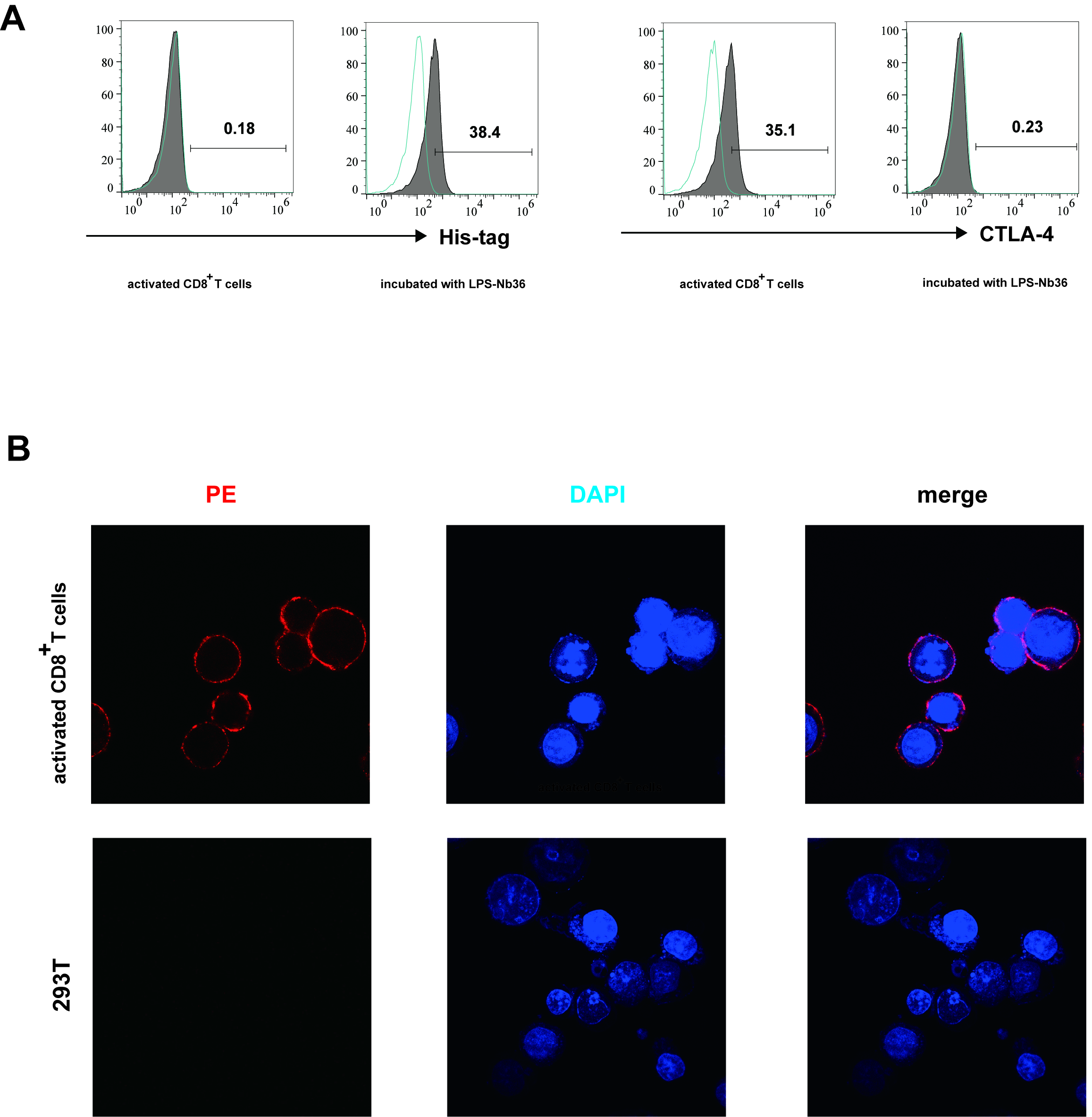

Supplement: Supplementary file 5 — Supplementary Figure s3 [file 41419_2023_5914_MOESM5_ESM.tif]

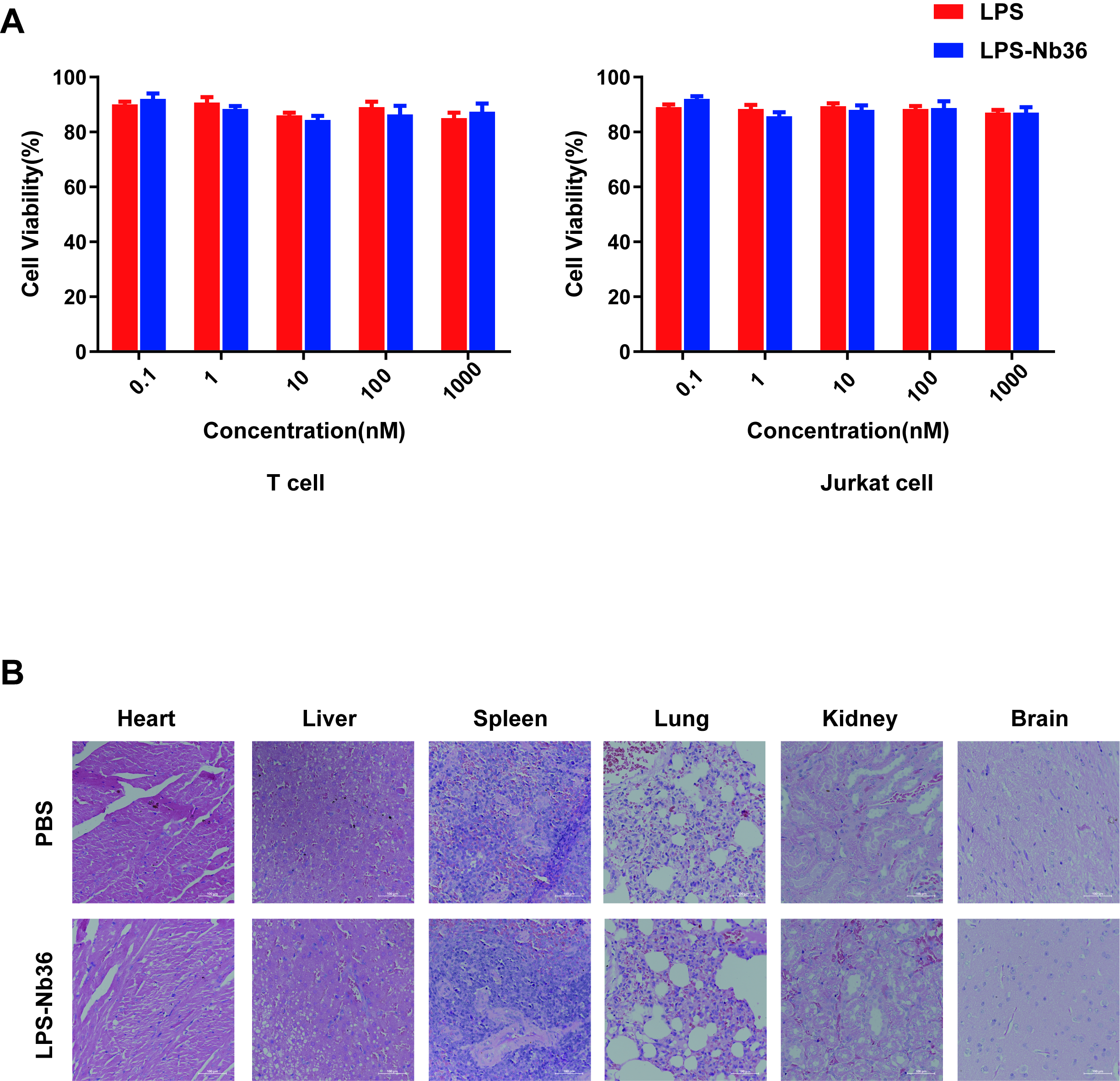

Supplement: Supplementary file 6 — Supplementary Figure s4 [file 41419_2023_5914_MOESM6_ESM.tif]

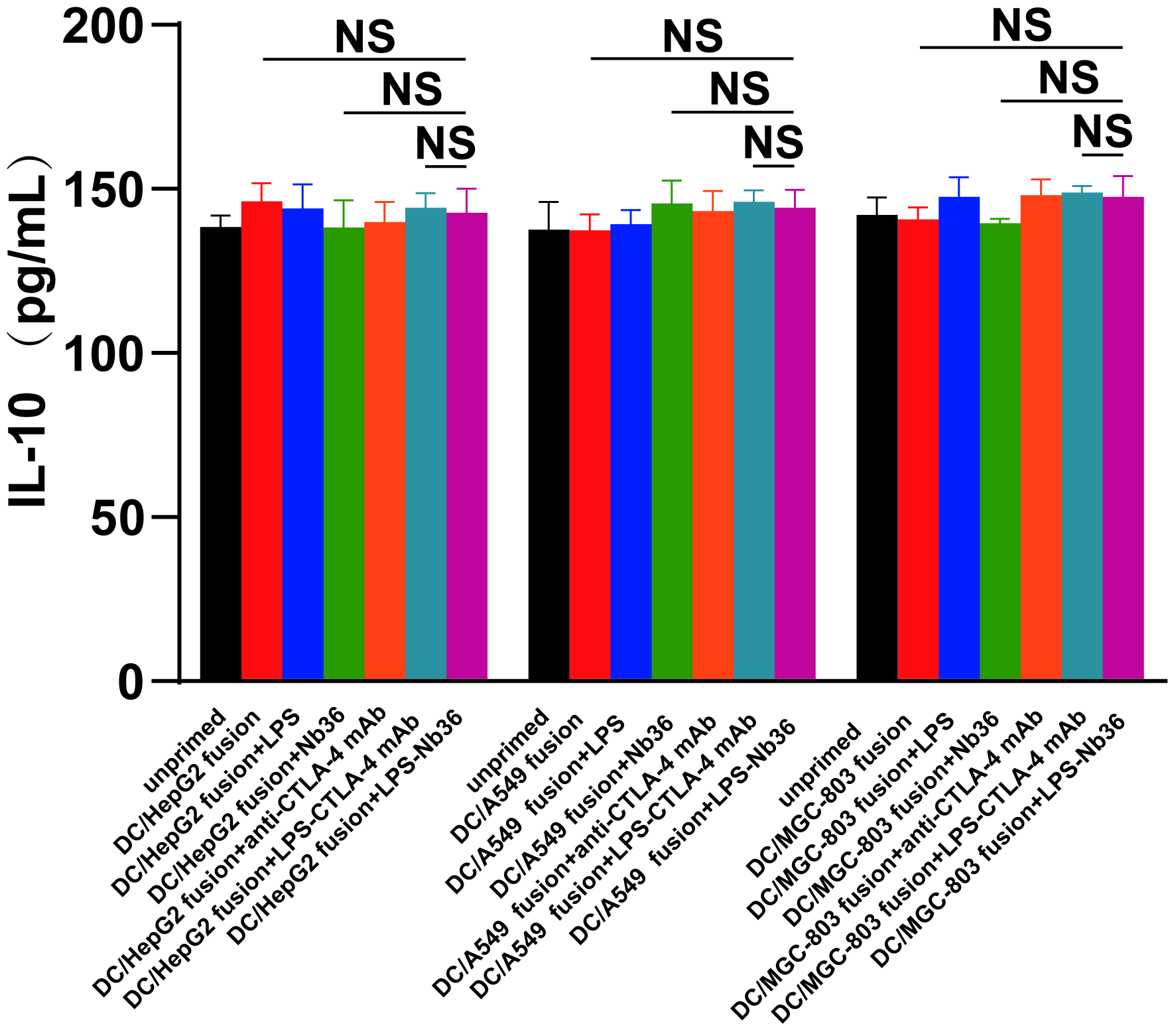

Supplement: Supplementary file 7 — Supplementary Figure s5 [file 41419_2023_5914_MOESM7_ESM.tif]

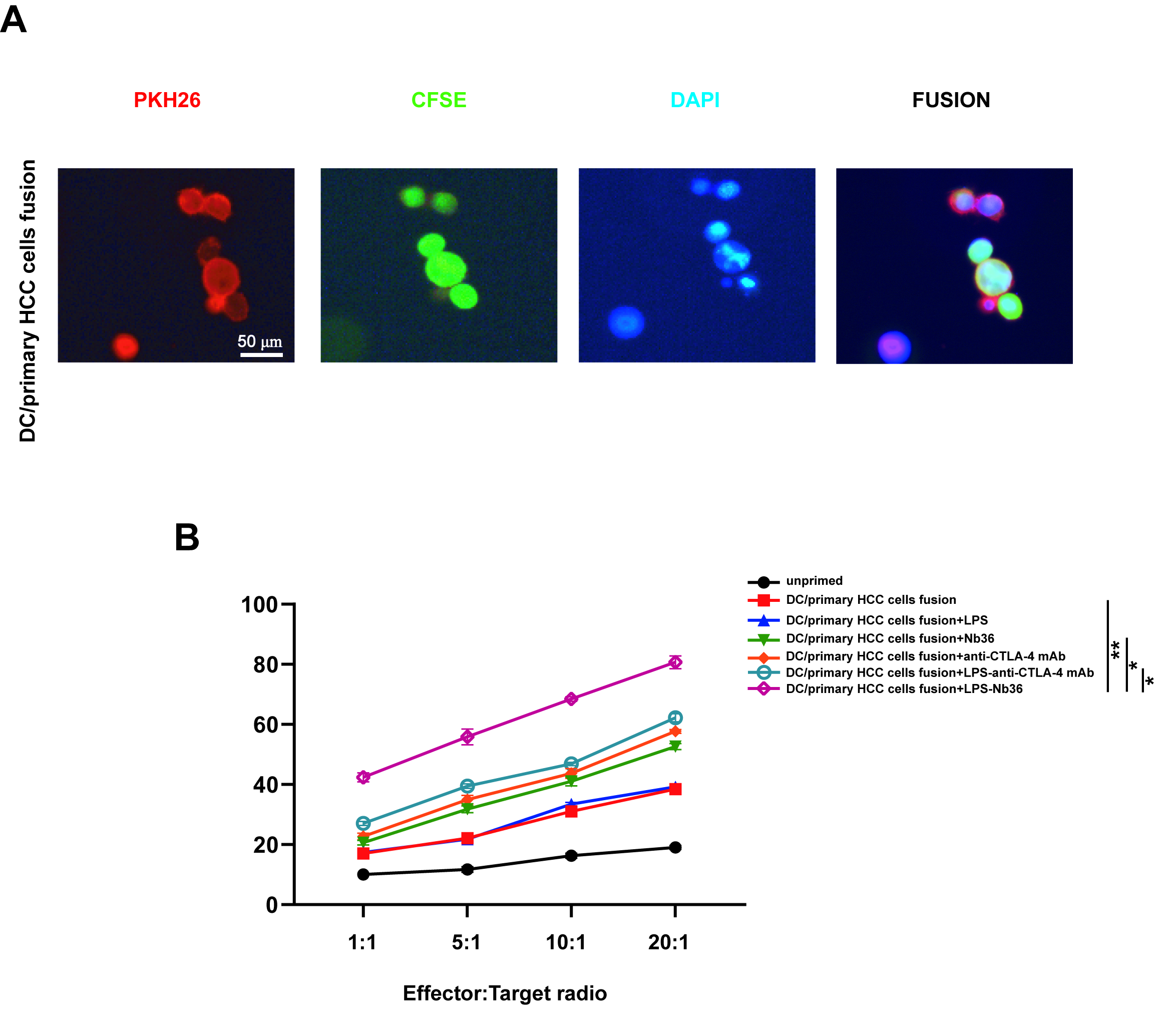

Supplement: Supplementary file 8 — Supplementary Figure s6 [file 41419_2023_5914_MOESM8_ESM.tif]
