# Supplementary material for: A novel CTLA-4 blocking strategy based on nanobody enhances the activity of dendritic cell vaccine-stimulated antitumor cytotoxic T lymphocytes
Source: Cell Death Dis. 2023 Jul 7;14(7):406. doi: 10.1038/s41419-023-05914-w (PMC10328924; doi:10.1038/s41419-023-05914-w)
Supplement: Supplementary file 9 — Supplementary table1 [file 41419_2023_5914_MOESM9_ESM.docx]

|  | Hydrodynamic size (nm) | PDI |
| --- | --- | --- |
| LPS | 112.45±0.80 | 0.197±0.003 |
| LPS-Nb36 | 118.18±2.64 | 0.201±0.002 |

Table1. The hydrodynamic size and polydispersity index(PDI) of LPS-Nb36.
